# Deep Brain Stimulation in KMT2B-Related Dystonia: Case Report and Review of the Literature With Special Emphasis on Dysarthria and Speech

**DOI:** 10.3389/fneur.2021.662910

**Published:** 2021-05-14

**Authors:** Maria Abel, Robert Pfister, Iman Hussein, Fahd Alsalloum, Christina Onyinzo, Simon Kappl, Michael Zech, Walter Demmel, Martin Staudt, Manfred Kudernatsch, Steffen Berweck

**Affiliations:** ^1^Department of Neurosurgery and Epilepsy Surgery, Spine- and Scoliosis Surgery, Schön Klinik Vogtareuth, Vogtareuth, Germany; ^2^Departmemt of Pediatric Neurology, Neuro-Rehabilitation and Epileptology, Schön Klinik Vogtareuth, Vogtareuth, Germany; ^3^Helmholtz Centre Munich, Institute of Neurogenomics, Neuherberg, Germany; ^4^Institute of Human Genetics, Technical University of Munich, Munich, Germany; ^5^Research Institute Rehabilitation, Transition, Palliation, Paracelsus Medical University, Salzburg, Austria; ^6^Dr. Von Hauner Children's Hospital, Ludwig-Maximilians- University Munich, Munich, Germany

**Keywords:** KMT2B-related dystonia, globus pallidus internus, internal capsule, dysarthria, case report, deep brain stimulation

## Abstract

**Objective:** KMT2B-related dystonia is a progressive childhood-onset movement disorder, evolving from lower-limb focal dystonia into generalized dystonia. With increasing age, children frequently show prominent laryngeal or facial dystonia manifesting in dysarthria. Bilateral deep brain stimulation of the globus pallidus internus (GPi-DBS) is reported to be an efficient therapeutic option. Especially improvement of dystonia and regaining of independent mobility is commonly described, but detailed information about the impact of GPi-DBS on dysarthria and speech is scarce.

**Methods:** We report the 16-months outcome after bilateral GPi-DBS in an 8-year-old child with KMT2B-related dystonia caused by a *de-novo* c.3043C>T (p.Arg1015^*^) non-sense variant with special emphasis on dysarthria and speech. We compare the outcome of our patient with 59 patients identified through a PubMed literature search.

**Results:** A remarkable improvement of voice, articulation, respiration and prosodic characteristics was seen 16 months after GPi-DBS. The patients' speech intelligibility improved. His speech became much more comprehensible not only for his parents, but also for others. Furthermore, his vocabulary and the possibility to express his feelings and wants expanded considerably.

**Conclusion:** A positive outcome of GPi-DBS on speech and dysarthria is rarely described in the literature. This might be due to disease progression, non-effectiveness of DBS or due to inadvertent spreading of the electrical current to the corticobulbar tract causing stimulation induced dysarthria. This highlights the importance of optimal lead placement, the possibility of horizontal steering of the electrical field by applying directional stimulation with segmented leads as well as the use of the lowest possible effective stimulation intensity.

## Introduction

Dystonia is a movement disorder characterized by abnormal and uncontrolled hyperkinetic movements as a result of sustained or intermittent muscle contractions (1). Dystonic symptoms can affect only one or several sites of the body, resulting in twisting and repetitive postures and movements (2). The etiology of dystonias is quite heterogeneous (3). With the advancement of next-generation sequencing techniques, several genetic causes of isolated and combined dystonia have been identified (4). Since 2016, several mutations in the *KMT2B* gene have been identified as a new etiology of early-onset dystonia (5–9). *KMT2B*-related dystonia is a progressive childhood-onset disorder, commonly evolving from a focal, mainly lower-limb dystonia into generalized dystonia with cranio-cervical involvement. Further clinical characteristics such as intellectual disability, psychiatric comorbidities and dysmorphic features have been reported in several patients with *KMT2B*-related dystonia (7). Bilateral deep brain stimulation of the globus pallidus internus (GPi-DBS) has been reported as an efficient therapeutic option. Especially improvement of the movement disorder and regaining of independent mobility is commonly described (8). Dysarthria is one of the most commonly described stimulation-induced side effects when GPi-DBS is used in dystonia (10). Nevertheless, detailed information about the impact of GPi-DBS on speech in *KMT2B*-related dystonia is scarce. Here, we report the 16-months outcome after bilateral GPi-DBS in an 8-year-old child with *KMT2B*-related dystonia with special emphasis on dysarthria and speech. We compare the outcome of our patient with 59 patients identified through a PubMed literature search.

## Case Report

A 6-year-old Arabic boy with generalized dystonia affecting the limbs, trunk, cervical muscles and facial muscles, resulting in severe disability in gait, speech and daily life activities was admitted to our inpatient department.

Pregnancy, perinatal, birth and early infantile history were unremarkable. Gross motor development was normal until the age of 3 years, when the child started limping. Few months later, he lost the ability to walk and reverted to crawling. After several years, dystonia also involved the cervical, oromandibular and laryngeal muscles, as well as both upper limbs. The patients' speech and language development were delayed: At the age of 18 months, the child could pronounce “dad” and “mum.” At the age of 2 years, the patient could speak two-word sentences in Arabic and English. Swallowing and chewing have not been affected by dystonia. The development of social and cognitive skills has also been delayed. Psychiatric comorbidities have not been reported.

On admission, the patient presented with already generalized dystonia and severe dysarthria. The child was wheelchair bound and sat unsteadily. Dystonia symptoms worsened at night, resulting in frequent nocturnal restlessness. Physical examination revealed intermittent myoclonus in both legs but no spasticity or other neurological symptoms. The patient showed developmental delay, a short stature (percentile: <3) and low body weight (percentile: <3). His face showed no dysmorphic features. The family history was negative for neurologic conditions. The Burke-Fahn-Marsden Dystonia Rating Scale (BFMDRS) score was 59 of 120 on the dystonia movement scale and 22 of 30 on the disability scale. Magnetic Resonance Imaging (MRI) of the brain and spine revealed no abnormalities. Also in retrospect, no symmetrical hypointensities of the globus pallidi commonly described in *KMT2B* mutations were present. Lumbar puncture to analyze neurotransmitters was denied.

Trio whole-exome sequencing, performed in a diagnostic laboratory had shown that the patient carried a heterozygous *de-novo* non-sense variant, c.3043C>T (p.Arg1015^*^), in *KMT2B* (11). According to American College of Medical Genetics & Genomics (ACMG) criteria, the variant was classified as “pathogenic.”

The patient was treated with retarded Carbidopa 25 mg / Levodopa 100 mg and Gabapentin 300 mg. The dystonic movement disorder did not improve significantly. The dystonia was generalized, and therefore injections of botulinum toxin were not performed.

After intensive rehabilitation including orthotic management and supplying the patient with a posterior walker, the child could walk a few steps (see Video 2 in the Supplementary Material). For longer distances a wheelchair was necessary.

**Figure 1:**
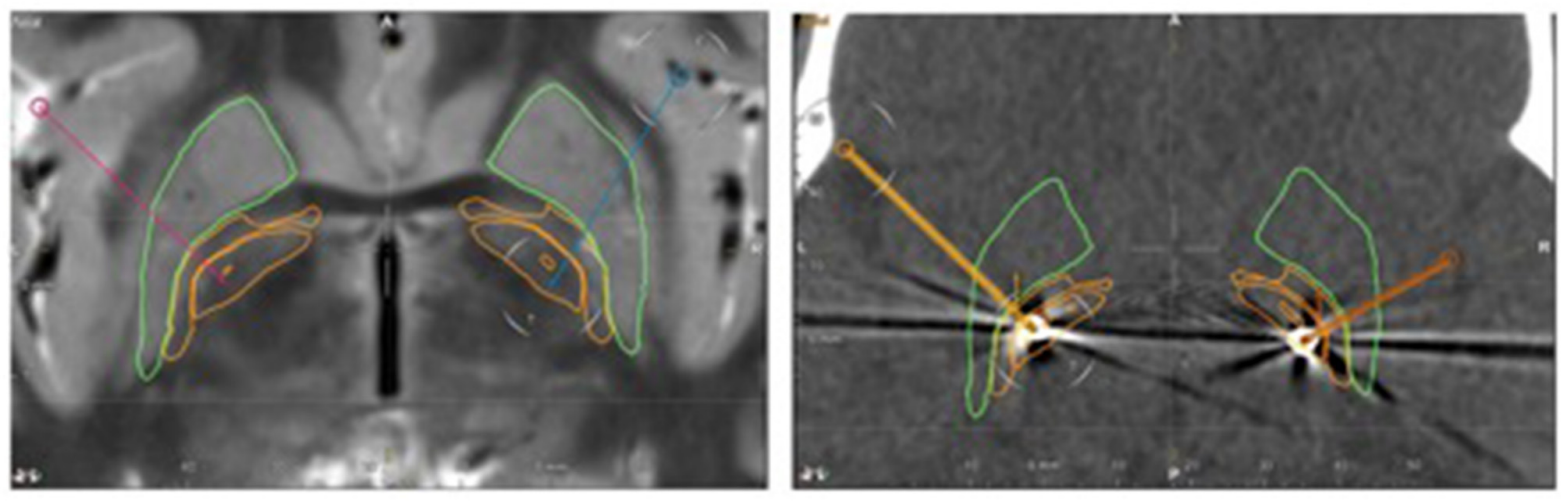
Left: Pre-operative axial T2-weighted MRI. Stereotactic planning performed with the Brainlab Elements software shows the planned trajectories (red and blue line). Right: postoperative axial CT. The pre-calculated electrode position is projected to the CT artifacts of the real electrode (yellow on the right side, orange on the left side). The leads are located within the caudal part of the GPi. Green outline: putamen, outer orange outline: globus pallidus externus, inner orange outline: globus pallidus internus.

After an extensive interdisciplinary discussion, GPi-DBS was performed at the age of eight years under general anesthesia. Stereotactic planning was done with the Brainlab Elements software, using MRI T1- and T2- sequences (T1 1 mm isotropic 3D with Gadolinium and T2 space 0.65 mm isotropic). The GPi could be visualized on the T2- images, as shown in Figure 1. Stereotactical electrode placement was done with a Leksell Multipurpose Arc G-Frame. Directional leads (Model Vercise TM Cartesia directional Lead, Boston Scientific), targeting the caudal part of the GPi were implanted successfully and a rechargeable as well as MRI-compatible impulse generator (Model Gevia Boston Scientific) was placed subcutaneously in the right infraclavicular region. Correct position of the leads was confirmed by postoperative computed tomography (CT) and image fusion to the planning scans in Brainlab Elements (see Figure 1).

Initially the amplitude was set to 2 mA on both sides with a pulse width of 80 μs and a frequency of 130 Hz. Due to the short attention span, a structured test by monopolar review at each contact was not possible. Therefore, we decided to start a non-segmental stimulation at contact 2–4 on the left and 10–12 on the right side. During the following weeks, we tested different amplitudes of 2–4 mA and pulse widths between 80 and 100 μs. A clinical improvement was already observed after several days, especially for motor symptoms of the arms and legs but speech did not improve at the same pace. During subsequent visits, we changed the programming at the right lead to a segmental stimulation with a current flow steering to a lateral direction (contact 10:25, 11:0, 12:75%). Parallel to this change the parents noticed a reduced stiffness in the left arm during active movements and a significant, but still variable improvement of speech fluency in addition to the already achieved amelioration of symptoms.

Six and sixteen months after surgery, the boy underwent a follow-up investigation and neurological rehabilitation. Six months after surgery, his father reported impressive improvement of dystonia. The child had already achieved independent walking. Nocturnal restlessness and involuntary motor movements had disappeared. On admission, the patient appeared more settled, confident and focused. His concentration, and consequently, his performance in activities of daily living had significantly improved. The BFMDRS score 6 months after GPi-DBS was considerably reduced, counting 28 of 120 (reduction of eight points in the categories “mouth/speech/swallowing” and 23 points in the categories “trunk/extremities”) on the dystonia movement scale and 11 of 30 on the disability scale.

An amelioration in speech and dysarthria after GPi-DBS was noticed. The modified Bogenhausen Dysarthria Scales as a clinical assessment with a defined scoring system (0 = most severe disorder; 1 = severe disorder; 2 = moderate disorder; 3 = mild disorder; 4 = no disorder) were applied before GPi-DBS as well as 6 and 16 months after surgery, by an Arabic speaking doctor and an Arabic speaking speech therapist. A moderate improvement from scale 1 to scale 2 was seen in articulation, voice quality, speech fluency and prosodic characteristics 6 months after surgery. A remarkable improvement from scale 1 to scale 3 was seen in articulation, voice quality, speech fluency and prosodic characteristics 16 months after surgery. The patients' speech intelligibility improved as well. His speech became much more comprehensible not only for his parents, but also for others. Furthermore, his vocabulary and the possibility to express his feelings and wants expanded considerably (Video 1 in the Supplementary Material shows the child counting in Arabic before and 16 months after GPi-DBS).

## Literature Review

We did not follow the methodology of a systematic review. We analyzed the cohort studies performed by Cif et al. (7) and Zech et al. (8) as these studies are the largest KMT2B-DBS cohorts reported to date. Further, we have performed a PubMed Search using the terms “*KMT2B*” and “*dystonia*” and Deep Brain stimulation.

We identified 15 studies including a total of 59 patients with *KMT2B*-related dystonia who underwent DBS between 2016 and 2020 (see Table 1 in the Supplementary Material).

In 56 cases, the GPi was targeted for lead implantation. In one case, the leads were implanted within the subthalamic nucleus. In two cases, lead placement was not described.

In 3/59 patients, improvement of speech (“articulation”/”dysphonia”/”orolingual dystonia”) was described (12–14). Amelioration of jaw opening dystonia was reported in 2/59 cases (5, 15). Persisting dysarthria/dysphonia after performance of DBS was described in 4/59 patients (6, 8, 13, 16). In 50/59 patients, the outcome of dysarthria and speech after DBS has not been specifically described.

Cif et al. reported a total of 18 patients with *KMT2B*-related dystonia who underwent GPi-DBS. In this study cohort, only 3.4 % of all patients showed benefit on speech at the last assessment (7). Carecchio et al. (9) reported eight patients with *KMT2B*-related dystonia, who underwent GPi-DBS. They observed laryngeal dystonia in some patients only after DBS was performed (9).

## Discussion

We report the 16-months GPi-DBS outcome of an 8-year-old child with *KMT2B*-related generalized dystonia.

Since 2016, mutations in the *KMT2B* gene have been identified in patients with early-onset dystonia (5–9). *KMT2B* encodes a lysine-specific histone methyltransferase, which is involved in an important methylation process for epigenetic modification linked to active gene transcription (17). Dysfunction or haploinsufficiency of *KMT2B* is assumed to affect the downstream expression of key genes that regulate neurodevelopment and motor control (7). *KMT2B*-related dystonia typically presents in childhood, commonly evolving from a lower-limb dystonia into generalized dystonia with cranio-cervical involvement (5–7). In our case, dystonia also started in the lower limbs, involving the cervical, oromandibular, and laryngeal muscles as well as the trunk and upper limbs a few years later. The median onset of age of *KMT2B*-related dystonia is reported to be 6 years, varying according to the subtype of *KMT2B* variants (17). In our patient, dystonia symptoms started at the age of 3 years. Facial dysmorphic features, developmental delay and intellectual disability are frequently observed in *KMT2B*-related dystonia patients as non-dystonic abnormalities (8, 17). Our patient showed development delay including short stature, low body weight and microcephaly as well as poor development of speech and language. There were no facial dysmorphic features.

Bilateral GPi-DBS is reported to be an overall efficient therapeutic option. Especially improvements of the movement disorder and regaining of independent mobility are commonly described (8). Oromandibular involvement of dystonia frequently results in disorders in speech and articulation in *KMT2B*-related dystonia (7–9). Furthermore, dysarthria additionally can occur as stimulation-induced side effect of GPi-DBS. A differentiation between dysarthria as a symptom of dystonia, regarding orofacial and buccolingual dystonia, that may or may not be responsive to GPi-DBS and dysarthria as a result of stimulation-induced side effect is necessary. In primary dystonia, the most common stimulation-induced side effect of GPi-DBS is reported to be dysarthria with a prevalence of up to 12 %. Dystonic patients who underwent DBS described a slowing of speech as well as requiring more effort for speech production even without clinically evident dysarthria (10). In terms of the established literature, studies with detailed information about the impact of GPi-DBS on dysarthria and speech in *KMT2B*-related dystonia are scarce. Speech disturbances can, however, severely impair the patients' quality of life. In only 3/59 patients in the literature, improvement of speech has been described (12–14). Nevertheless, persisting dysarthria / dysphonia after performance of DBS has been reported in 4/59 patients (6, 8, 13, 16). In addition, Carecchio et al. observed no improvement or even newly developing laryngeal dystonia in some *KMT2B* patients only after DBS was performed (9). The underlying cause is not described, but probably related to laryngeal dystonia associated with the progression of disease.

In this case report, the patient presented with orofacial dystonia, which was responsive to GPi-DBS. Further, a stimulation-induced side effect resulting in dysarthria did not occur. Dysarthria as well as delayed speech and language development were identified and assessed before performance of GPi-DBS in our case. According to the modified Bogenhausen Dysarthria Scales, a remarkable improvement of voice, articulation, respiration and prosodic characteristics were found 16 months after GPi-DBS. Furthermore, the patient's vocabulary and the possibility to express his feelings and wants expanded considerably after surgery.

Up to now, little is known about possible mechanisms of stimulation-induced changes in speech associated with GPi-DBS. A possible neuroanatomical mechanism is described as an inadvertent spreading of the electrical current to the corticobulbar tract in the adjacent internal capsule that represents the face region somatotopically (18). Therefore, higher stimulation intensities as well as more posterior location of active lead contacts could result in stimulation-induced dysarthria associated with undesirable spreading of the stimulation to the corticobulbar tract in the adjacent internal capsule (10).

Stimulation settings, as well as specific positioning of the leads into the GPi, have not been described in the cases with improvement of speech after GPi-DBS identified in our literature review. We assume that the positive effects on dysarthria and speech in our case are achieved by low stimulation intensity, avoiding stimulation of the corticobulbar tract by lead positioning within the caudal part of the GPi, and horizontal steering of the electrical field, using the features of the directional DBS-leads. A standardized protocol to test directional vs. non-directional stimulation would be useful for every patient, but in the majority of our pediatric patients and in the patient reported in this case report, the compliance does not allow this lengthy procedure.

Moreover, our patient seemed to have a huge potential for speech development, which he could not take advantage of because of the severity of the dystonia. Therefore, minimizing dystonia-related disorders regarding speech as well as movement disturbances led to impressive amelioration in speech after GPi-DBS implantation.

One important limitation of our case report is the short follow-up after GPi-DBS. We take into consideration that our patient was very young at time of surgery. Performing GPi-DBS surgery is a symptomatic treatment. The course of disease cannot be influenced. Therefore, the disease can progress despite ongoing efficient DBS therapy. As *KMT2B*-related dystonia is a progressive disease, a long-term follow-up is necessary. A decrease and even loss of stimulation effect as well as disease progression in our patient could occur in the future.

## Conclusion

Our case report demonstrates an improvement of dysarthria and speech after GPi-DBS in an 8-year old boy with *KMT2B*-related generalized dystonia.

A positive outcome of GPi-DBS on speech and dysarthria is rarely described in the literature. This might be due to disease progression, non-effectiveness of DBS or due to inadvertent spreading of the electrical current to the corticobulbar tract causing stimulation induced dysarthria. This highlights the importance of optimal lead placement, the possibility of horizontal steering of the electrical field by applying directional stimulation with segmented leads as well as the use of the lowest possible effective stimulation intensity. Future studies should implicate specific speech and articulation outcome assessments before and after performance of GPi-DBS associated with *KMT2B*-related dystonia as well as long-term outcomes after GPi-DBS.

## Data Availability Statement

The original contributions presented in the study are included in the article/Supplementary Materials, further inquiries can be directed to the corresponding author/s.

## Ethics Statement

Ethical review and approval was not required for the study on human participants in accordance with the local legislation and institutional requirements. Written informed consent to participate in this study was provided by the participants' legal guardian/next of kin. Written informed consent was obtained from the minor(s)' legal guardian/next of kin for the publication of any potentially identifiable images or data included in this article.

## Author Contributions

MA and SB planned the article concept and design. RP and WD performed the surgery planning. WD and RP performed the surgery. IH and FA conducted the modified Bogenhausen Dysarthria Scales. MA performed the literature review. MA wrote the manuscript with support from RP, MZ, MS, and SB. All authors discussed the results and contributed to the final manuscript.

## Conflict of Interest

WD serves as a teacher of implantation techniques to Boston Scientific, Inc. and receives compensation for these services. The remaining authors declare that the research was conducted in the absence of any commercial or financial relationships that could be construed as a potential conflict of interest. The handling Editor declared a shared affiliation, though no other collaboration, with one of the authors MZ.
