# Supplementary material for: Deep Brain Stimulation in KMT2B-Related Dystonia: Case Report and Review of the Literature With Special Emphasis on Dysarthria and Speech
Source: Front Neurol. 2021 May 14;12:662910. doi: 10.3389/fneur.2021.662910 (PMC8160374; doi:10.3389/fneur.2021.662910)
Supplement: Supplementary file 1 [file Table_1.DOCX]

*Table 1 All studies and case reports that reported on patients with KMT2B-related dystonia who underwent DBS surgery in the period between 2016 and 2020.*

| **Study** | **DBS cohort (n)** | **Speech Outcome descriptions after DBS (original descriptions from the literature indicated with quotation marks)** | **Target of electrodes** | **Additional information (original descriptions from the literature indicated with quotation marks)** |
| --- | --- | --- | --- | --- |
| Cif et al., 2020 (1) | n=17  (n=18; Patient 59 has been already described with effects on DBS surgery by Meyer et al.,2016;  Patient 54-58 have not been described in regard with DBS surgery in Meyer et al.,2016) | “At the last assessment, BFMDRS-D scores were variable, improvement maintained for swallowing (52.9%), dressing (40.0%) and writing (40.0%), whilst benefit on gait (16.2%) and speech (3.4%) were suboptimal.” | all targeted the GPi |  |
| Li et al., 2020 (2) | n=8  (n=9; Patient 5 has been reported by Cao et al.,2020) | (Patient 5:  “Orolingual, limb and truncal dystonia improved significantly”)    Patient 18:  „articulation improved dramatically“ | 8 targeted the GPi, 1 targeted the subthalamic nucleus (STN) |  |
| Miyata et al., 2020 (3) | n=1 | no data. | targeted the GPi | Before DBS was performed:  „His speech and swallowing were not affected “ |
| Mun et al., 2020 (4) | n=1 | no data. | targeted the GPi |  |
| Carecchino et al., 2019 (5) | n=8 | “laryngeal dystonia did not show relevant improvement regardless of stimulation parameters, and some patients even developed laryngeal dystonia after DBS” | all targeted the GPi |  |
| Meyer et al., 2017 (6) | n=10 | Patient 19:  “Improvement of jaw-opening dystonia and tongue protrusion” | all targeted the GPi | Before DBS was performed:  Patient 19:  “Anarthria”, “Jaw-opening dystonia”, “Tongue protrusion”,  “swallowing difficulties”,  “Severe speech delay” |
| Kawarai et al., 2018 (7) | n=3 | Patient A:  “The dysartrophonia was also improved with increased voice volume.”  Patient B:  “The dysphonia remains unimproved”  Patient C:  “Dysphonia and scoliosis remained unimproved.” | all targeted the GPi | Before DBS was performed:  Patient A: “reached normal developmental milestones by the age of 5.”; “Dysartrophonia was evident later.”  Patient C: “speech and language learning was also delayed” |
| Dafsari et al., 2019 (8) | n=2 | no data. | all targeted the GPi | Before DBS was performed:  Patient #1:  “Dysarthria”; “dysphonia” |
| Garrido et al., 2018 (9) | n=1 | “excellent improvement of symptoms, recovery of independent walking, and partial recovery of writing and manual dexterity, however dysarthria persisted” | targeted the GPi | Before DBS was performed:  “severe disability on speech” |
| Zhao et al., 2018 (10) | n=1 | no data. | no data. |  |
| Zech et al., 2016 (11) | n=1 | Patient F1-II-5:  “The last follow-up examination at age 31 years showed some residual degrees of abnormal neck and trunk posturing, along with persisting dystonic gait impairment and dysarthric speech.” | all targeted the GPi | Before DBS surgery was performed:  Patient F1-II-5:  “In subsequent years, dystonic symptoms progressed to involve other body areas including the face, neck, larynx, tongue, trunk and all four extremities.” |
| Zech et al., 2017 (12) | n=2 | no data. | all targeted the GPi |  |
| Zech et al., 2019 (13) | n=1 | Patient F4-III2:  no data. | targeted the GPi |  |
| Cao et al., 2020 (14) | n=1 | “Orolingual, cervical truncal, and limb dystonia improved significantly” | targeted the GPi | Before DBS was performed:  “He reached all developmental milestones until age 7 when he first experienced impairment of speech” |
| Kumar et al., 2019 (15) | n=2 | Pt.9125:  “good improvement of jaw opening, cervical and upper limb dystonia”  Pt.17106:  no data. | Pt.9125:  no data  Pt.17106:  targeted the GPi | Before DBS was performed:  Pt.9125:  “he developed dystonia of the left foot and laryngeal dystonia by five years of age”  Pt.17106:  “later evolved to severe dysarthria with oromandibular dystonia” |

References:

1. Cif L, Demailly D, Lin JP, Barwick KE, Sa M, Abela L, et al. KMT2B-related disorders: expansion of the phenotypic spectrum and long-term efficacy of deep brain stimulation. Brain. 2020;143(11):3242-61.

2. Li XY, Dai LF, Wan XH, Guo Y, Dai Y, Li SL, et al. Clinical phenotypes, genotypes and treatment in Chinese dystonia patients with KMT2B variants. Parkinsonism Relat Disord. 2020;77:76-82.

3. Miyata Y, Hamanaka K, Kumada S, Uchino S, Yokochi F, Taniguchi M, et al. An atypical case of KMT2B-related dystonia manifesting asterixis and effect of deep brain stimulation of the globus pallidus. Neurol Clin Neurosci 2020; 8: 36–8.

4. Mun JK, Kim AR, Ahn JH, Kim M, Cho JW, Lee J, et al. Successful pallidal stimulation in a patient with KMT2B-related dystonia. J Mov Disord 2020; 13: 154–8.

5. Carecchio M, Invernizzi F, Gonzàlez-Latapi P, Panteghini C, Zorzi G, Romito L, et al. Frequency and phenotypic spectrum of KMT2B dystonia in childhood: A single-center cohort study. Mov Disord. 2019;34(10):1516-27.

6. Meyer E, Carss KJ, Rankin J, Nichols JM, Grozeva D, Joseph AP, et al. Mutations in the histone methyltransferase gene KMT2B cause complex early-onset dystonia. Nat Genet. 2017;49(2):223-37.

7. Kawarai T, Miyamoto R, Nakagawa E, Koichihara R, Sakamoto T, Mure H, et al. Phenotype variability and allelic heterogeneity in KMT2B-Associated disease. Parkinsonism Relat Disord. 2018;52:55-61.

8. Dafsari HS, Sprute R, Wunderlich G, Daimagu¨ler H-S, Karaca E, Contreras A, et al. Novel mutations in KMT2B offer pathophysiological insights into childhood-onset progressive dystonia. J Hum Genet 2019; 64: 803–13.

9. Garrido A, Simonet C, Martí MJ, Pérez-Dueñas B, Rumià J, Valldeoriola F. Good response to bilateral GPI-DBS after 2 years in generalized dystonia due to a mutation in the KMT2B gene (DYT28) [abstract]. Mov Disord. 2018;33(suppl 2) https://www. mdsabstracts.org/abstract/good-response-to-bilateral-gpi-dbs-after2-years-in-generalized-dystonia-due-to-a-mutation-in-the-kmt2bgene-dyt28/. Accessed 14 June 2019. 19.

10. Zhao K, van der Spoel A, Castiglioni C, Gale S, Fujiwara H, Ory DS, et al. 19q13.12 microdeletion syndrome fibroblasts display abnormal storage of cholesterol and sphingolipids in the endolysosomal system. Biochim Biophys Acta Mol Basis Dis. 2018;1864(6 Pt A):2108–18. https://doi.org/10.1016/j.bbadis. 2018.03.020. 37.

11. Zech M, Boesch S, Maier EM, Borggraefe I, Vill K, Laccone F, et al. Haploinsufficiency of KMT2B, Encoding the Lysine-Specific Histone Methyltransferase 2B, Results in Early-Onset Generalized Dystonia. Am J Hum Genet. 2016;99(6):1377-87.

12. Zech M, Jech R, Havra´ nkova´ P, Fecı´kova´ A, Berutti R, Urgoı´k D, et al. KMT2B rare missense variants in generalized dystonia. Mov Disord 2017a; 32: 1087–91.

13. Zech M, Lam DD, Winkelmann J. Update on KMT2B-Related Dystonia. Curr Neurol Neurosci Rep. 2019;19(11):92.

14. Cao Z, Yao H, Bao X, Wen Y, Liu B, Wang S, et al. DYT28 responsive to pallidal deep brain stimulation. Mov Disord Clin Pract 2020; 7: 97–9.

15. Kumar KR, Davis RL, Tchan MC, Wali GM, Mahant N, Ng K, et al. Whole genome sequencing for the genetic diagnosis of heterogenous dystonia phenotypes. Parkinsonism Relat Disord. 2019;69:111-8.
